# Brain tissue development of neonates with Congenital Septal Defect: Study on MRI Image Evaluation of Deep Learning Algorithm

**DOI:** 10.12669/pjms.37.6-WIT.4863

**Published:** 2021

**Authors:** Jianfei Zhu, Jiaolei Chen, Yunhui Zhang, Jianwei Ji

**Affiliations:** 1 Jianfei Zhu, Attending Physician. Department of Neonatology, Yiwu Central Hospital, Yiwu, 322000, China; 2 Jiaolei Chen, Attending Physician. Department of Neonatology, Yiwu Central Hospital, Yiwu, 322000, China; 3 Yunhui Zhang, Attending Physician. Department of Neonatology, Yiwu Central Hospital, Yiwu, 322000, China; 4 Jianwei Ji, Attending Physician. Department of Neonatology, Yiwu Central Hospital, Yiwu, 322000, China

**Keywords:** Brain Tissue, Neonates, Congenital Septal Defect, MRI Image Evaluation, Deep Learning Algorithm

## Abstract

**Objectives:**

This article is based on deep learning algorithms and uses MRI to study the development of congenital heart septal defects in neonatal brain tissue.

**Methods:**

From January 2018 to December 2019, 150 cases of congenital cardiac paper septal defect were retrospectively analyzed on 50 cases of normal newborns and neonates. The four index parametersbrain MR imaging, lateral ventricle pre-angle measurement index (F/F’), body index (D/ D’), caudal nucleus index (C/C’) were analyzed. The independent sample t test is performed to compare the difference parameters between groups.

**Results:**

F congenital heart disease group and control group/F ‘values were 0.301 ± 0.035 and 0.296 ± 0.031; Evans index was 0.239 ± 0.052 and 0.233 ± 0.025; 2 sets of D/D’ values were 0.261 ± 0.039 and 0.234 ± 0.032; C/C ‘value was 0.138 ± 0.018 and 0.124 ± 0.015 respectively. The congenital heart disease group D/D ‘, and the value of C/C’ obtained under the ROC curve area value, respectively 0.698 and 0.750, Youden index corresponding to the maximum D/D ‘, and the value of C/C’ values were 0.28 and 0.12.

**Conclusion:**

Lateral ventricle D/D ‘and C/C’ is more sensitive indicator which can be evaluated with the difference between the volume of congenital heart septal defects in newborn normal neonatal brain; when the D/D ‘value> 0.28, C/C’ value> 0.12. For the diagnosis and evaluation of congenital heart septal defect neonatal brain volume abnormalities have a certain reference value.

**List of acronyms:**
MRI: Magnetic Resonance Imaging.
POX: Pulse oximetry.
CHD: Congenital Heart Disease
DWI: Diffusion-weighted Imaging.
T1WI: T1-weighted imaging
T2WITSE: T2-weighted imaging, Turbo Spin Echo
FOV: Field of View
FLAIR: Fluid Attenuated Inversion Recovery
TE: Echo Time
TR: Repetition Time
ICC: Intra-group Correlation Coefficient

## INTRODUCTION

Pulse oximetry (POX) has been widely used in clinical practice. It has become a supplement to prenatal ultrasound and postnatal clinical feature evaluation.^1^-^3^ The corresponding sensitivity is about 75%, and the specificity is about 99.9%.^4^,^5^ Case-control methods explore the prenatal related factors in children with CHD (Congenital heart disease) in combination with previous studies, and collect clinical data to evaluate the sensitivity of different initial symptoms in children with CHD and the impact of early diagnosis and treatment.^6^,^7^

This study intends to use MRI technology to objectively quantify the developmental status of brain tissue, achieve a preliminary assessment of the brain development status of newborns with CHD, and discover possible early brain development abnormalities in newborns with CHD.

## METHODS

We covered 150 newborns (CHD group) in our hospital from January 2018 to December 2019. There were 82 males and 68 females. One hundred and twenty-six had atrial septal defect, 15 had ventricular septal defect, and 9 had atrial septal defect with ventricular septal defect. All newborns underwent head MRI scans. 50 newborns in the control group were clinically diagnosed as “neonatal pneumonia (except severe pneumonia)” or “scalp mass”, and the intracranial imaging findings by MRI were negative. The average gestational age was (295±11) days and the average body weight was (3091±615) g. There were 29 males and 21 females.

### Exclusion criteria

Gestational age <38 weeks or >42 weeks, multiple births, congenital neurological defects not related to congenital septal defect, chromosomal abnormalities or syndromes, brain infections, hydrocephalus, hypoxic ischemic encephalopathy, etc.

Siemens 1.5 Tavant MR scanner is used, and the head orthogonal coil was selected. All newborns had the same standard head MRI scan: sagittal T1WI (T1-weighted imaging) sequence (TE9ms, TR4440ms, reversal angle 150°, FOV180mm×180mm, slice thickness 4.5mm); transverse T2WITSE(T2-weighted imaging, Turbo Spin Echo) sequence (TE117ms, TR5570ms, reverse Rotation angle 150°, FOV180mm×180mm, layer thickness 4.5mm); transverse position T2WI FLAIR sequence (TE92ms, TR6000ms, reversal angle 150°, FOV180mm×180mm, layer thickness 4.5mm); transverse position DWI(Diffusion-weighted Imaging) sequence (TE99ms, TR3200ms, reverse The angle of rotation is 150°, and the b value was 0s/mm2, 800s/mm2).

To ensure the smooth progress of the examination and to obtain the ideal image, 5% chloral hydrate 1mL/kg was given oral for sedation before the neonatal MRI scan, and the blood oxygen saturation and electrocardiogram were closely monitored during the examination.

After the acquisition was completed, two physicians measured four lateral ventricle indexes on the obtained cranial MR images: (1) Lateral ventricular anterior horn index, the distance between the outermost end of the lateral ventricle anterior horn and the same level and the same horizontal brain transverse diameter Ratio F/F’; (2) Lateral ventricle body index, the ratio of the smallest diameter of the outer edge of the lateral ventricle body to the same level and the same level of the brain transverse diameter D/D’.

### Deep learning algorithm for image segmentation

The relationship between contrast and brightness can be expressed as a function:


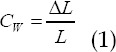


∆L is the difference between the brightness of the target object and the background brightness L. From the analysis of human visual physiology, the human eye has the characteristics of multi-frequency channel decomposition, and the interaction between the channels makes humans have different effects on the recognition of things in different resolution environments. In the multi-channel visual characteristics, human vision has a masking effect. If the spatial frequencies between the two signals are very similar, the masking effect is more obvious.

When facing a complex scene, the human eye will quickly lock on several obvious targets. Because the targets are different, the introduction of visual attention characteristics into the computer vision system requires the construction of a filter model.

The conditions should ensure that there is no overlap between the segmented areas, the same segmented area has one or more attributes, and the pixels in the same segmented area can also have the attribute characteristics of other areas.^8^ The quality of segmentation can be evaluated by using human visual characteristics, which can be evaluated through the subjective perception of the human eye, and mathematical models.

**Fig.1:**
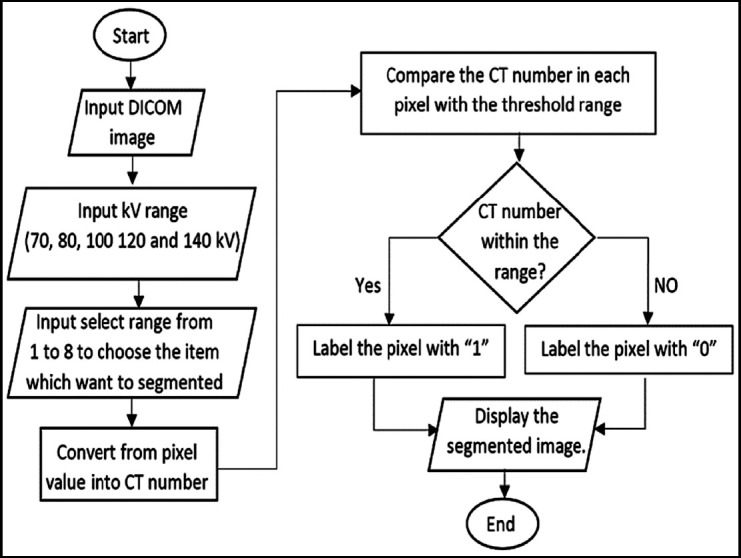
Threshold segmentation algorithm flow.

The threshold segmentation method separates the target from the background image by calculating the threshold, and generates a binary image to simplify the analysis and processing process.^9^ It is very useful for motion detection, optical recognition, image defect repair, etc. Fig.1 shows the threshold segmentation algorithm flow.

However, the traditional threshold segmentation method is based on image gray characteristics and spatial information. The basic principle is to set the original image as f, the image size as *M × N*, and the gray level number as L. *f(x,y)* is *the* coordinate in the image as *(x,y)*, The number of pixel gray levels, 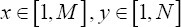, determine a segmentation threshold T to segment all pixel gray levels, and get the result:


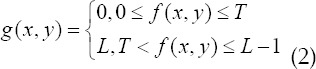


If the segmentation threshold T is applicable to the entire image, then T is the global threshold, and if it applies to a local area, it is called a local threshold. If multi-threshold segmentation is used, the number of thresholds is set to n, the segmentation threshold is expressed as *T*_1_,*T*_2_,...,*T*_n_, ,and the segmentation result obtained is expressed as:


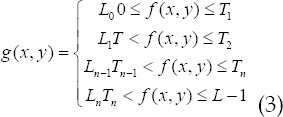


The local threshold segmentation method is to divide a large image into multiple sub-regions to calculate local features.^10^ Some researchers mix the global threshold segmentation method with the local threshold segmentation method and use the advantages of the global and local threshold method to segment. However, it is still impossible to perform a certain amount of calculation.

This study integrates the visual characteristics of the human eye into the segmentation of graphics and images, constructs a method for segmentation of graphics and images, assigns a visual objective function to the threshold, and optimizes the function, calculates the adaptive threshold of the image local attributes, and then obtains the segmentation. The human eye is very sensitive to changes in local edge contrast. Therefore, gradient information is used to reflect the gray changes of the edge of the image.^11^ The Sobel operator is used to detect the edge. To reduce the influence of background noise, a classification function is defined, and the function is expressed as:


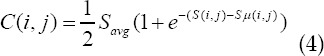


*S_avg_* is the average gray value of the gradient image, *S(i,j)* is the gradient value and *Sμ(i,j)* is the gradient average of the 3×3 local window. The gray value of the pixel is compared with the value of the classification function to determine the classification label of the pixel. The amount of binary image data generated by threshold segmentation is small. Otsu uses the maximum inter-class difference method to construct a sub-image containing the main detail information and a sub-image containing the remaining information from the original image. The sub-image of information is expressed:


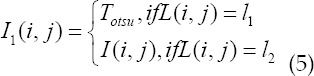


*I_1_* is the sub-image that contains the main detail information, *I_2_* is the sub-image that contains the remaining information, *T_otsu_* is the global threshold obtained by the Otsu method, *L(i,j)* is the classification label of the pixel *(i,j)*, and *l*_1_,*l*_2_ is the classification label.

The global optimal thresholds *T*_1_ and *T*_2_ of images *I*_1_ and *I*_2_ can be obtained, and the segmentation results *B*_1_ and *B*_2_ can be obtained. Each binary image contains part of the original gray-scale image, let B be the final two of image I Value map, for pixel 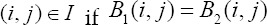 gets:


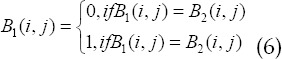


### Statistical Analysis

Using SPSS19.0 statistical software package, all measurement results are expressed as mean ± standard deviation, independent two-sample t-test (single-tailed) is used for differences between groups, χ^2^ test is used for count data, and α=0.05 is used as the significance standard of difference; The non-parametric receiver operating characteristic curve (ROC curve) analysis method was used to obtain the possible positive diagnostic cut-off points of the disease group for the different ventricular index. The reliability of the data measured by the two measurers is evaluated using the intra-group correlation coefficient (ICC), 0 means unreliable, one means completely reliable. If the data results of two testers are credible, take the result of tester one as the final calculation result.

## RESULTS

The ICC values of F/F’, D/D’, C/C’, and Evans index measured by two measurers were 0.872, 0.837, 0.796, 0.880, respectively. The reliability of the two determinations is high, and the measurement consistency of this method is high as well.

The CHD group and the control group had no statistically significant differences in gestational age, birth weight, and gender (t=0.159, P=0.874; t=0.568, P=0.500; χ^2^=0.870, P=0.350). The CHD group had no significant difference in F/F’, Evans index measurement values (P>0.05), while the CHD group D/D’ and C/C’ measurement values were larger than the normal group (P<0.05, Table-I).

**Table-I:** MRI parameter measurement results of CHD group and control group.

	*CHD group*	*Control group*	*t*	*P*
F/F’	0.301±0.035	0.296±0.031	1.035	0.302
D/D’	0.261±0.039	0.234±0.032	3.873	0.003*
C/C’	0.138±0.018	0.124±0.015	4.479	0.000*
Evans Index	0.239±0.052	0.233±0.025	0.778	0.438

## DISCUSSION

In this study, 200 newborns were selected as the research objects, 150 of which were CHD in the CHD group, and 50 in the control group were “neonatal pneumonia” or “scalp mass”. Both groups of patients underwent the same head MRI scan, and the neonatal brain MRI images were processed with an adaptive threshold segmentation algorithm. Then, F/F’, D/D’, C/C’, Evans index, etc. were taken to evaluate brain MRI images and analyze the developmental characteristics of the brain tissue of the two groups of children, and excellent examination results were obtained. The results showed that there was no statistical difference in the basic data between the children in CHD group and the control group. Through testing F/F’, D/D’, C/C’, Evans, and other indexes, it was found that there was no significant difference in F/F’ (0.301±0.035 vs 0.296±0.031) and Evans index (0.239±0.052 vs 0.233±0.025) between the two groups of patients (*P*>0.05), which means CHD won’t cause the change of the maximum width/maximum double parietal diameter of the frontal angle of the lateral ventricle and the frontal angle of the same plane. This was consistent with the research results of Limperopoulos et al.^12^ Therefore, it was believed that there was no significant correlation between neonatal F/F’ and Evans index and neonatal cardiac septal defect. Heart septal defect did not cause changes in newborn F/F’ and Evans index. However, the two indexes of D/D’ (0.261±0.039 vs 0.234±0.032) and C/C’ (0.138±0.018 vs 0.124±0.015) were significantly different between the two groups (*P*<0.05), which was similar to the research results of Feldmann et al.^13^ D/D’ is the body index of the lateral ventricle of the brain, C/C’ is the index of the caudate nucleus of the brain. The difference of D/D’ and C/C’ between the CHD group and the control group indicated that the brain volume of children with CHD was relatively small. This was also consistent with the reduced brain volume in children with complex CHD that had been confirmed by earlier studies.^14^,^15^ In addition, it was found that there was a certain correlation between D/D’, C/C’, and the size of the cardiac septal defect in children. This difference may be used in clinical studies of children with congenital cardiac atrial defect.

Non-parametric ROC was used to further analyze the two indexes of D/D’ and C/C’, and it was found that the area under the curve of the D/D’ and C/C’ values of the CHD group were 0.698 and 0.750, respectively, and the corresponding D/D’ and C/C’ when the Youden index was the largest were 0.28 and 0.12, respectively. The ROC curve can reflect the ability of a certain diagnostic method to distinguish the diseases.^16^ There was a significant difference between the two groups of patients when D/D’ and C/C’ were greater than 0.28 and 0.12, respectively, which suggested that D/D’ and C/C’ can be used as indexes of brain development in children with congenital atrial septal defect. The use of adaptive threshold segmentation to evaluate brain MRI images of children had certain guiding significance for the development of brain tissue in congenital atrial septal defect. The detection of lateral ventricle body index and caudate nucleus index was of certain clinical value in monitoring the development of brain tissue in children with CHD.

## CONCLUSION

In summary, this study shows that there is a certain delay in the development of brain tissue in children with CHD. D/D’ and C/C’ can be used as a simple and sensitive method to monitor the development of brain tissue in children with CHD.

### Authors’ Contribution:

**JZ:** Conceived the study, literature review, data analysis, drafting the manuscript. **JC & YZ:** Helped in design, data collection, drafting & critical revision. **JJ:** Takes the responsibility and is accountable for all aspects of the work in ensuring that questions related to the accuracy or integrity of any part of the work are appropriately investigated and resolved.
